# Supplementary material for: Proinflammatory Polyphosphate Increases in Plasma of Obese Children with Insulin Resistance and Adults with Severe Type 2 Diabetes
Source: Nutrients. 2022 Nov 1;14(21):4601. doi: 10.3390/nu14214601 (PMC9654964; doi:10.3390/nu14214601)
Supplement: Supplementary file 1 [file nutrients-14-04601-s001.zip › nutrients-1990940-supplementary.pdf]

Supplementary Materials
Montilla et al., 2022.

Figure S1.

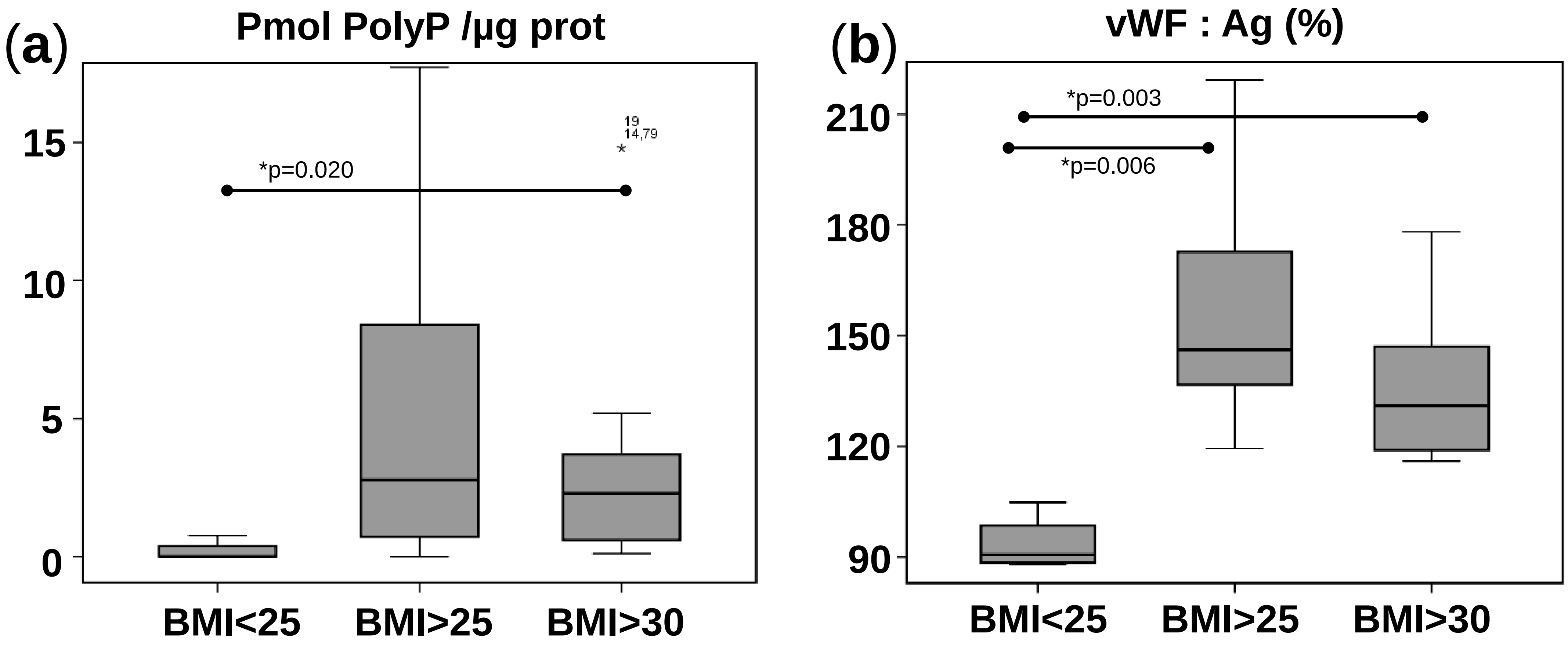

Figure S1. (**a**) Plasma polyphosphate (polyP) levels, from individuals described in Table 1. There are significant differences (p=0,050), determined by Kruskal-Wallis test. (**b**) Plasma von Willebrand factor antigen (vWF:Ag), from individuals described in Table 1. There are significant differences (p=0,007), determined by Kruskal-Wallis test. Results are presented in a box-and whiskers plot and the asterisks indicate a statistical difference of p<0.05, determined by Mann–Whitney test.

Supplementary Materials
Montilla et al., 2022.

Figure S2.

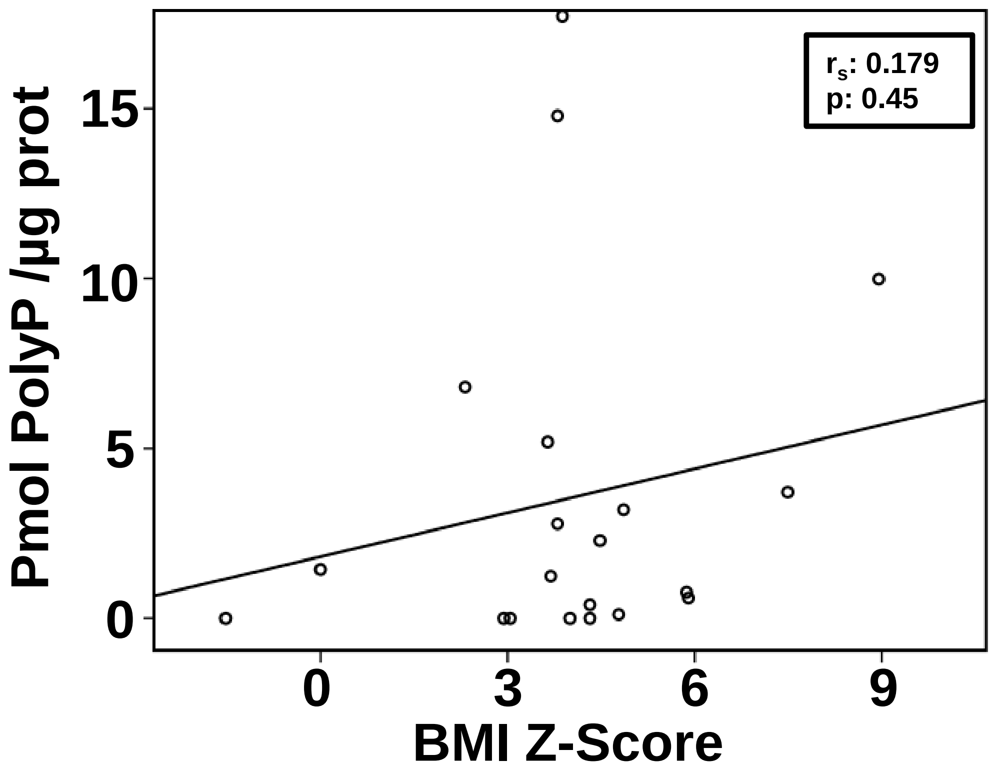

Figure S2. Graphic representation and Spearman's correlation analysis of BMI Z-score and levels of plasma polyP of all individuals measured in Figure 1. "r_s": Spearman's correlation coefficient.

Supplementary Materials
Montilla et al., 2022.

Figure S3.

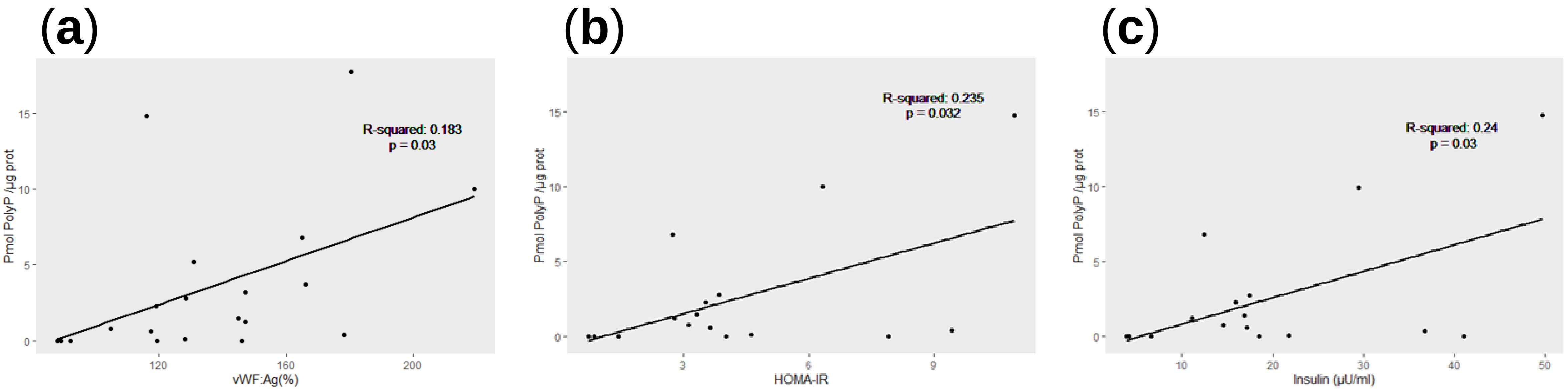

Figure S3. Simple linear regression plots of plasma polyP and variables, from the list in Table 2, that have significant correlations: (**a**) vWF; (**b**) iHOMA; (**c**) Insulin.
